# National Medical Union Notes

**Published:** 1913-12-27

**Authors:** E. Arthur Dorrell, R. Carswell

**Affiliations:** Hon. Sec.; Hon. Sec.


### National Medical Union Notes.

The Executive Committee met in London on 20th inst., Dr. Greenyer in the chair. Provisional arrangements were made for the official publication of National Medical Union news in The Hospital, and for the circulation of that paper amongst the whole of the members. Any member who does not receive a copy of The Hospital is requested to communicate with one of the Hon. Secretaries. The Secretaries were instructed to proceed at once to take suitable offices, preferably at the *Morning Post* Buildings, 346 Strand, or in some other central locality. Various sub-committees were appointed to proceed actively with the business of the Union, and the meeting was encouraged by the evidence forthcoming that its policy has won the active support of a large number of practitioners throughout England and Scotland. Applications for membership are coming in with encouraging and continuous volume.

E. Arthur Dorrell and R. Carswell,
Hon. Secs.

